# Identification of Chemical Composition and Metal Determination of *Retama raetam* (Forssk) Stem Constituents Using ICP-MS, GC-MS-MS, and DART-MS

**DOI:** 10.1155/2021/6667238

**Published:** 2021-02-24

**Authors:** Wedad Al-Onazi, Amal M. Al-Mohaimeed, Musarat Amina, Maha F. El-Tohamy

**Affiliations:** ^1^Department of Chemistry, College of Science, King Saud University, Riyadh 11451, Saudi Arabia; ^2^Department of Pharmacognosy, College of Pharmacy, King Saud University, Riyadh 11451, Saudi Arabia

## Abstract

This study aims to investigate the chemical constituents of the stem of *Retama raetam* growing in Saudi Arabia. The organic and inorganic composition of ethanol extract of *R. raetam* stem has been explored using direct analysis in real time-mass spectrometry (DART-MS), gas chromatography-mass spectrometry (GC-MS), and inductively coupled plasma-mass spectrometry (ICP-MS). Analysis conducted by DART-MS and GC-MS reveals the presence of several interesting organic constituents identified as 2,4-di-tert-butylphenol, sparteine, benzenepropanoic acid, 3,5-bis(1,1-dimethylethyl)-4-hydroxy-, methyl ester, phthalic acid, 1-octadecanol, squalene, argentamin, 2,4-di-tert-butylphenol, sparteine, benzene propanoic acid, 3,5-bis(1,1-dimethylethyl)-4-hydroxy-, methyl ester, phthalic acid, 1-octadecanol, squalene, argentamin, and hentriacontane in the ethanol extract of the *R. raetam* stem. The ICP-MS analysis of stem extract showed the presence of a significant amount of important inorganic elements including aluminum, chlorine, calcium, bromine magnesium, phosphorus, scandium, and chromium. The current study complements other *R. raetam* extract investigations carried out in the past and provides the additional data for the future research studies.

## 1. Introduction

Aromatic and medicinal plants are considered as the primary natural source for a wide variety of volatile terpene, hydrocarbons, essential oils, and secondary metabolites. Essential oils also known as volatile oil are being widely used in traditional medicine. Among others, antibacterial, antifungal, antirheumatic, and anti-inflammatory activities have been reported in the literature for these essential oils [1–3]. Genus *Retama* is comprised of monophyletic taxon belonging to family Fabaceae. It is comprised of four structurally related species distributed from endemic to the Mediterranean area: *R. sphaerocarpa* (L.) Boiss., *R. dasycarpa* Coss., *R. monosperma* (L.) Boiss., and *R. raetam* (Forssk.) Webb [4, 5]. *R. raetam* is commonly found in the Sinai Peninsula and North East Mediterranean region. The extracts of aerial parts of various species of genus *Retama* are used as traditional remedies in treatment of hypertension, diabetes, rheumatism, and anti-inflammatory diseases [6–8]. Moreover, several studies in the literature report different pharmacological properties of *Retama* species which includes antioxidant, antibacterial, antifungal, hepatoprotective, antiproliferative, antiulcerogenic, hypoglycemic, antihypertensive, and diuretic effects [9–16]. Phytochemical investigation has shown that *Retama* species are rich in flavonoids, quinolizidine, and bipiperidyl alkaloids and the cyclitol pinitol compounds [17–21]. *Retama raetam* (Forssk) Webb, commonly known as ‘R'tm, is the most valuable aromatic and medicinal wild plant. It is naturally grown in the northeastern Mediterranean region, the Sinai Peninsula, Tunisia, Libya, and Saudi Arabia [22, 23]. The flowers of this plant emit sweet honey aroma, and roots are deeply rooted in the soil. It has slender branches which reduces the exposed surface area to make plants resistant to dry conditions of the desert. The leaves are very small and quickly drop to conserve the water. Traditionally, different parts of plants are used in the treatment of renal and skin diseases. Its extracts are reported to possess potential diuretic activity and could be useful in the cure of hypertension. *R. raetam* have also shown antidiabetic effect. In Tunisia, it is found in abundance and is being used for various industrial purposes. In Algeria and Morocco, it is used to reduce the blood glucose and skin inflammation [24, 25]. However, in Saudi Arabia, the plant is used for wound healing and inflammation. Also, cattle fed with *R. raetam* made their milk sweeter.

A plenty of scientific data have been generated in the use of traditional medicines in the past two decades mainly encompassing the natural product-based drugs. Hundreds of reports are available in the literature on plant components, their isolation, identification, and studies related to their pharmacological and toxicological properties. Most of the scientific studies of natural products are concerned towards the identification and characterization of organic components, which are mostly secondary metabolites. Indeed, organic compounds are the responsible components of the biological activities. However, the inorganic elements are also present in the plant extracts, and they could play a potential influential role in the therapeutic effects of the natural products. Inorganic components can take part as supplementary materials in addition to the medicinal effects of herbal-based medicines [26, 27].

A number of studies addressed the organic constituents of *R. raetam*; however, the inorganic elements are not reported yet. The current work emphasizes on the organic as well as inorganic components of the *R. raetam* stem extract using DART-MS, GC-MS, and ICP-MS techniques.

## 2. Materials and Methods

### 2.1. Plant Material

The stems of plant *Retama raetam* (2.5 Kg) were collected from the Mada'in salehh, North west of Saudi Arabia, in October 2019. The plant material was authenticated and identified by a taxonomist at the Botany Department of King Saud university. The dried stems were coarse powdered in a mixer grinder to get the uniform size. The powdered material of *R. raetam* was transferred to polyethylene plastic polybags and kept in a cold place at 4°C, until further use.

### 2.2. DART-MS Analysis

Direct analysis in real-time study was performed in AccuTOF-DART-MS, JEOL (Inc., Peabody, MA, USA), equipped with an ionic source DART-SVPTM (Ionsense, Saugus, MA, USA). Stem sample of the botanical plant material was directly sampled by holding with forceps between the DART ion source and the mass spectrometer. The detection of chemical constituents by this technique does not require the extraction or sample preparation. The volatile components of the stem are evaporated in a stream of helium heated at 350°C and were then ionized by excited metastable helium atoms prior to entering the ion source of the mass spectrometer. The molecules are mainly protonated without any fragmentation in a positive ionization mode.

#### 2.2.1. DART-MS Instrumental Parameters

All mass measurements were performed using DART-AccuTOF mass spectrometer in a positive ion mode. Protonated reserpine was used to measure the resolving power of spectrometer at 6000 (FWHM). The mass spectrum of polyethylene glycol (1500 average molecular weight) was chosen as a reference standard for exact mass measurements of each generated compound in the plant sample. The following conditions for the atmospheric pressure interface were applied: ring lens (4 V); orifice 1 (10 V) and orifice 2 (5V). The voltage of radio frequency (RF) ion guide was adjusted at 800 V to permit detection of ions greater than *m/z* 80. Helium gas was used as a DART ion source at the 350°C with a flow rate 2 L/min and a grid voltage of 530 V, and input gas pressure was adjusted to 1.8 × 10^−2^ Pa (Airgas, Cambridge, MA). Elemental compositions of chemical constituents detected were compared with accurate mass spectral data with well-developed library of plant components isolated from the NIST standard reference database (2008 database).

### 2.3. Preparation of *R. raetam* Extract for GC-MS Analysis

To analyze the organic components *R. raetam* plant, powdered stem (100 g) was subjected to ethanol extraction. The plant powder was soaked in 300 mL of ethanol for 48 h, at ambient temperature. The ethanolic extract was drained, filtered, centrifuged, and freed from solvent on the rotavapour at ±50°C, under reduced pressure to get the dark brown residue (16.5 g). The obtained extract was stored in the refrigerator at 4°C in dark air-tight bottles prior to further analysis with GC-MS.

#### 2.3.1. GC-MS Instrumental Conditions

The chemical composition of the ethanol extract obtained from the stem of *R. raetam* was analyzed in a Thermo Trace GC ultra-gas chromatography coupled with a TSQ quantum mass spectrometer (triple quadrupole), available at College of Science, King Saud University. 2 *μ*l solution of stem extract of *R. raetam* was loaded into the glass capillary column fused with Elite-5MS column (30 × 0.25 mm internal diameter with 0.25 mm × 0.25 *μ*m film thickness). The stationary phase is 5% phenyl polysilphenylene-siloxane. The temperature of the oven was set at 40°C, 5 min, reaching 300°C at an acceleration rate of 10°C/min. The injector and detector temperature was maintained at 250°C. Helium was used as the carrier gas with a flow rate of 0.5 mL and a split flow of 25 mL/min, corresponding to split ratio of 50. The total acquisition time for analysis was 75 min. Mass detector scanning was carried out at 40 to 500 (m/z), in an ionization mode. The mass detector was operated at 70 eV ionization energy and 0.132 s/scan in the full scan mode. Relative abundance (% area) calculations were based on the ratio between the peak area of each compound and the sum of the peak areas of all compounds [28]. National Institute of Standard and Technology (v2.1, NIST, 2005, Wiley) library was used to identify the unknown components and the comparison with literature reported.

### 2.4. Preparation of Sample for ICP-MS Analysis by Microwave Digestion

The dried, powdered plant material (515 mg) was placed in dry, clean Teflon microwave digestion vessel, to which 2.0 mL of concentrated HNO_3_, 6.0 mL of HCl, and 2.0 mL of HF were added. The sample was then subjected to digestion using scientific microwave (Model Milestone Ethos 1600) at 165°C for 10 min with the microwave irradiation of 1000 W power, followed by a dwell time 20 min at 165°C. The temperature and pressure limit was 175°C and 15.2 bar (220 psi), respectively. After cooling, the resulting digest was transferred to a 50 mL plastic volumetric flask and made up to the mark using deionized water. A blank digest was carried out in the same way.

#### 2.4.1. ICP-MS Instrumental Conditions

For the trace elemental analysis of *R. raetam,* the instrument NexION 300D (inductively coupled plasma-mass spectrometer, PerkinElmer, USA) was used to conduct the ICP-MS analysis.  Table 1 highlights the operating conditions of the instruments used in this study.

#### 2.4.2. Calibration of ICP-MS and Internal Standard

Instrument calibration was performed using 1.0 ppb multielement internal standard solution in 1% nitric acid of various elements such as lithium (^6^Li), beryllium (^9^Be), zinc (^67^Zn), selenium (^78^Se), bromine (^81^Br), cobalt (^59^Co), sodium (^23^Na), magnesium (^24^ Mg), silicon (^28^Si), iron (^57^Fe), chromium (^53^Cr), lead (^208^Pb), copper (^63^Cu), copper (^65^Cu), and barium (^136^Ba). The same standard solution was applied to optimize the gas flow, mass calibration, resolution, and AutoLens calibration. For all analyses, 20 ppb multielement internal standard solution was used.

### 2.5. Bacterial Strains

Five different pathogenic bacterial strains were used in this study including *Salmonella typhi* ATCC, *Escherichia coli* ATCC, *Pseudomonas aeruginosa* ATCC, and *Klebsiella pneumoniae* ATCC as Gram-negative and *Staphylococcus aureus* ATCC as Gram-positive bacterial strain. The bacterial strains were supplied by the King Khalid Hospital, Saudi Arabia, and were used to investigate the antibacterial potential of ethanol extract of *R. raetam.* Various physiological, morphological, and biochemical tests were conducted to identify the selected bacterial strains [29]. All bacterial isolates were tested for antibacterial susceptibility by modifying the Kirby–Bauer disc diffusion method following the Clinical and Laboratory Standards Institute (CLSI) guidelines [30].

### 2.6. Antibacterial Assay of *R. raetam* Extract

Agar well diffusion method was used to investigate the antibacterial potential of ethanol extract of *R. raetam* stem in Mueller–Hinton Agar (MHA) plates [31]. The test bacterial strains were placed in nutrient broth and incubated for 12 h at 37°C to make the turbidity to 0.5 McFarland standards yielding a final inoculum of 1.5 × 108 CFU/mL. The standardized bacterial culture was spread on MHA plate. 50 mg·mL^−1^ solution of plant extract was prepared in dimethyl sulfoxide (DMSO). A sterile cork-borer was used to bore the inoculated media into the wells, and 50 *μ*L of plant extract was added to each well. Streptomycin was used as positive control, while DMSO was used as a negative control. The mixture was allowed to diffuse for approximately 30 min at ambient temperature and incubated at 37°C for one complete day. After the incubation time, the plates were evaluated for clear zone formation around the well, which expresses the antibacterial potential of the tested plant extract. The zone of inhibition was measured in mm.

### 2.7. Determination of MIC of the Plant Extract

MIC was determined by using a broth microdilution procedure according to the instruction of CLSI. Microorganisms were cultured in nutrient broth for 6 h. 20 mL of the cultured bacterial strains were inoculated in the tubes containing nutrient broth supplemented with seven different concentrations (20, 40, 80, 160, 320, 640, and 1280 mL) of the plant extract (25 mg mL^−1^) and incubated for one day at 37°C. The MIC of the sample was determined by measuring the optical density using spectrophotometer at 620 nm. Chloramphenicol was used as a standard substance [32].

### 2.8. DPPH Free Radical Scavenging Activity

The antioxidant effect of *R. raetam* stem extract was determined spectrophotometrically using the DPPH method by modifying previously described Brand-Williams et al. [33]. The method was conducted by mixing 1.5 mL of 20 *μ*g mL^−1^ of DPPH solution with 0.75 mL of 100 *μ*g mL^−1^ of plant extract. The sample under investigation was mixed well and kept in a dark at ambient temperature for 30 min. The absorbance of the mixture was measured spectrophotometrically at 517 nm against blank solution (0.75 mL of water mixed with 1.5 mL DPPH) using UV-Vis spectrophotometer (Shimadzu, Kyoto, Japan). Furthermore, to eliminate the crude extract absorbance, a blank sample was prepared by mixing 0.75 mL of plant extract with 1.5 mL of methanol. Thus, the antioxidant capacity percentage was calculated using the following equation:

(1) $$\begin{array}{c} \text{antioxidant activity }\left(\%\right)=\left[\frac{\left(A_{\text{Control}}-A_{\text{Extract}}\right)}{A_{\text{Control}}}\right]\times 100, \end{array}$$

where *A*_Control_ and *A*_Extract_ are the absorbance of the DPPH solution without the extract and absorbance of the tested plant extract with DPPH, respectively.

## 3. Results and Discussion

### 3.1. Chemical Analysis of *R. raetam* Stem Extract by DART-MS, GC-MS, and ICP-MS

**Figure 1:**
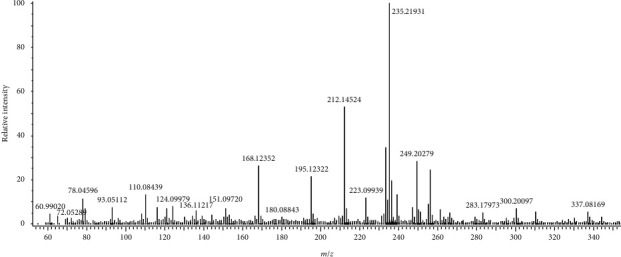
Main constituents characterized in *R. raetam* plant stem by DART-MS.

**Figure 2:**
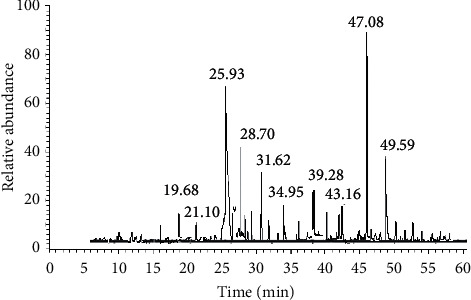
GC-MS-MS chromatogram of ethanol stem extract of *R. raetam*.

In the current study, phytochemical evaluation of organic and inorganic components of *R. raetam* stem extract were identified and quantitatively estimated by DART-MS, GC-MS, and ICP-MS analysis. A significant quantity of 22 organic chemical components was determined using DART-MS technique (Table 2).  Figure 1 displays the mass spectrum of organic compounds by the DART-MS, whereas 12 organic constituents were estimated by GC-MS technique (Table 3).  Figure 2 illustrates the chromatogram of organic compounds of *R. raetam* stem extract by GC-MS. However, a total of 33 inorganic constituents were detected using ICP-MS technique (Table 4). The results revealed that a significant amount of important organic compounds identified in the *R. raetam* stem extract include adiponitrile, 2-isopropylimidazole, cinachyrazole C, cadalene, 8-hexylisoquinoline, sparteine, 2-dodecyl-1H-imidazole, and 4-methyl-2-undecylimidazole in DART-MS analysis. Also, the GC-MS results showed the presence of valuable organic constituents, including 2,4-di-tert-butylphenol, sparteine, benzene propanoic acid, 3,5-bis(1,1-dimethylethyl)-4-hydroxy-, methyl ester, phthalic acid, 1-octadecanol, squalene, argentamin, and hentriacontane

Sparteine is a heterobicyclononane alkaloid known to possess antiarrhythmic effect, to decrease the incidences of ventricular tachycardia and fibrillation, and helps in reduction of blood pressure and heart rate [34, 35]. It also showed a hypoglycemic effect and induces glucagon and insulin secretions in the pancreas [36, 37]. Other biological properties which are reported for this alkaloid include anti-inflammatory, antibacterial, and diuretic effects and induce uterine contractions [38, 39].

GC-MS analysis estimated and identified the presence of hentriacontane in the stem extract of *R. raetam* (Table 3). Hentriacontane is reported to possess anti-inflammatory effects on the lipopolysaccharide- (LPS-) induced inflammatory responses in mouse peritoneal macrophages. It has inhibited the production of interleukin (IL-6), tumor necrosis factor (TNF-*α*), and prostaglandin-E2 (PGE 2), suggesting a potential candidate for the development of new inflammatory drugs to cure inflammatory diseases [40].

Antibacterial, antifungal, and cytotoxicity activities of *R. raetam* extract has been reported in the literature [10], which revealed that the constituents present in the plant have a major role in biological properties. A study conducted on a flower extract of *R. raetam* showed strong antimicrobial activity against *P. aeruginosa, E. coli*, and *Candida* species (7.81–15.62 *μ*g mL^−1^). The tested plant extract as well as flavonoids isolated showed strong cytotoxicity against Hep-2 cells [10].

Hentriacontane also known as untriacontane is a long chain hydrocarbon belonging to organic class alkanes. It is commonly found in a variety of food items such as saffron, sweet cherry coconut, and swamp cabbage. This makes a potential biomarker for the consumption of these food products. It has been reported for various pharmacological properties including, antitumor, anti-inflammatory, and antimicrobial activities [41].

Reactive oxygen species (ROS) are known to be responsible for oxidative cellular macromolecules damages such as nucleic acids, lipids, and proteins. These types of biological destruction may lead to disease progression and cell death. The stem extract of *R. raetam* displayed potent DPPH radical scavenging activity. The results attributed this activity due to the presence of terpenoids and flavonoids in the plant extract [42]. All of these addressed features of the *R. raetam* could be taken into account to support its medicinal applications in a traditional folk system of medicine [43]. In the current work, appreciable amount of sesquiterpenoids was detected in the *R. raetam* stem extract.

Inorganic components play a vital role in the survival of the bioactive chemical entities. In addition to four basis building elements hydrogen, carbon, oxygen, and nitrogen (forming main organic molecules), various inorganic components are required by the living organisms for their healthy survival. Several elements are essentially required for normal physiological body functions in humans [44]. In the current analysis, a total of 33 inorganic elements were detected in the *R. raetam* stem extract (Table 4), suggesting that they have an important role in biological functions in mankind.

The concentration of calcium was found to be the highest in the sample followed by iron, potassium, and sodium. Calcium is a vital element for numerous physiological functions and a structural material for bone in combination with phosphorus. Intake of calcium supplement helps in preventing bone fracture and calcium deficiency disorders [45]. Calcium was determined in the highest amount (71951 ± 3.1 mg Kg^−1^) among all the inorganic elements identified in the tested plant in the present study. Other potential nutrient elements found in the extract were iron, potassium, and sodium (Table 4). Iron is an important element which helps in oxygen carrying capacity of hemoglobin. It is also found in various important enzymes such as cytochrome p450 enzyme. The amount of iron recorded in *R. raetam* stem extract was 1054.487 ± 1.0 mg Kg^−1^ (Table 4).

Potassium, which was also found in higher quantity in the *R. raetam* extract (15267.514 ± 0.9 mg Kg^−1^), is reported to participate in regulating fluid balance, nerve signals, and muscle contraction. Studies have shown that high-potassium diet helps in reducing blood pressure and water retention, prevent osteoporosis and kidney stones, and protect against stroke.

Sodium is one of the useful elements required in small amounts via food to perform the normal biological processes. It helps to conduct nerve impulses, maintain the proper balance of water and minerals, and contraction and relaxation of muscles. The daily requirement of sodium by the human body is 500 mg. In the current investigation, 531.674 ± 2.4 mg Kg^−1^ of sodium was detected in the *R. raetam* extract. Higher levels of sodium intake have been negatively correlated with the risk of high blood pressure, heart disease, and stroke. Other significant trace nutrient elements detected in the *R. raetam* extract were phosphorus, strontium, manganese, and chromium (Table 4). Phosphorus is considered as an important component of bones, teeth, DNA, and RNA. It plays a crucial role in the structure of cell membrane and a key source of body's energy. Several sugars and proteins present in the body are phosphorylated. In this study, phosphorus has been found in an appreciable amount (701.293 ± 2.1 mg Kg^−1^).

The results of the current investigation revealed that four elements other than essential ones caught the attention for their substantial quantity in the *R. raetam* stem extract. These include bromine (35.389 ± 0.9), barium (15.622 ± 1.0 mg Kg^−1^), copper (6.648 ± 0.7 mg Kg^−1^), and arsenic (1.400 ± 2.4 mg Kg^−1^). These inorganic elements are toxic in nature. Table 3 also demonstrates the other elements identified in trace amounts.

### 3.2. Evaluation of Antibacterial Potential of *R. raetam* Stem Extract

Antibacterial activity of *R. raetam* stem extract was determined using agar well diffusion method against five different bacterial strains. The plant extract used in this study showed a varying degree of antibacterial potential against all the bacterial strains used. The potency of plant extract was measured qualitatively as well as quantitatively to access the presence or absence of zone of inhibition, zone diameter, and MIC values. Streptomycin and DMSO were used as positive and negative control, respectively. The data presented in Table 5 show that the ethanol extract of *R. raetam* stem has excellent antibacterial activity against all tested bacterial strains. The results revealed that the highest zone of inhibition was recorded against *P. aeruginosa* (6.2 mm) and *S. aureus* (5.8 mm), whereas the streptomycin gave inhibition zone between 1.65 and 3.74 mm. The ethanol extract of *R. raetam* showed a MIC of 2 and 5 mg mL^−1^ against *P. aeruginosa* and *S. aureus*, respectively. However, chloramphenicol had varied MIC from 6.5 to 10.5 mg mL^−1^. The results obtained in the present study supports with the previous study conducted by Awen et al. (2011) [46].

### 3.3. Evaluation of Antioxidant Potential of *R. raetam* Stem Extract

**Figure 3:**
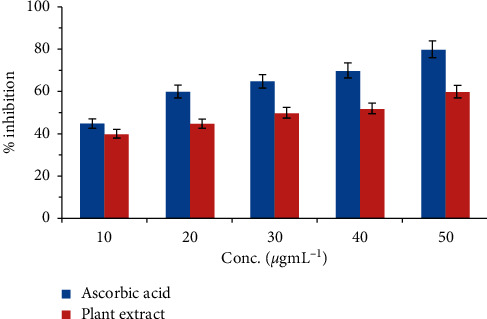
Results of DPPH radical scavenging potential of the *R. raetam* stem in comparison with ascorbic acid as reference.

The antioxidant potential of *R. raetam* stem extract was evaluated using DPPH free radical scavenging assay. It is considered as one of the simplest, effective, reliable, reactive, and reproducible *in vitro* procedures used for the evaluation of pure compound's activity as well as a plant extract. The results of DPPH radical scavenging potential of the *R. raetam* stem was compared with reference (ascorbic acid) and are presented in Figure 3. The measured antioxidant potential was estimated as IC_50_ values of DPPH free radical scavenging activity exhibiting strong antioxidant activity for the ethanol extract of *R. raetam* stem. The IC_50_ values obtained were 32.6 and 12.8 *μ*g mL^−1^ for the extract and ascorbic acid, respectively. The free radical scavenging abilities of the ethanol plant extract were lower as compared to ascorbic acid. The obtained results indicated that *R. raetam* stem extract has a strong hydrogen-donating ability, can act as a free radical scavenger, and can serve as possible substitute primary antioxidant. The strong antioxidant potential of *R. raetam* stem extract can be attributed to the presence of secondary metabolites.

## 4. Conclusion

This study provides an insight about the bioactive organic metabolites as well as beneficial inorganic elements of ethanol extract of *R. raetam* stem. It is probably the first report regarding the inorganic elements of the *R. raetam* extract and clearly supports the traditional use of *R. raetam* in the folk, traditional system of medicine. As a whole, *R. raetam* extract contains essential inorganic components needed for biological functions and traces of mercury, arsenic, and lead toxic elements. The outcome of present investigation offers the excellent role of the inorganic constituents in the medicinal properties together with its organic components. It also indicated the possibility of using this plant as a source of supplements for various inorganic elements in case of deficiency.

## Figures and Tables

**Table 1:** Instrument operating conditions for the determination of metallic species in *R. raetam*.

Instrument operating conditions	
R power	1600 W
Nebulizer gas flow	0.92 L/min
Lens voltage	9.25 V
Analog stage voltage	−1762.5 V
Pulse stage voltage	1050 V
Number of replicates	3
Reading/replicates	20
Scan mode	Peak hopping
Dwell time	40 ms
Integration	1200 ms

**Table 2:** The main constituents characterized in *R. raetam* plant stem by DART-MS.

S. no.	Experimental mass	Calculated mass	Mass difference (mmu)	Formula	Proposed name	Unsaturation degree
1	108.06872	108.06875	−*0.03*	C_6_H_8_N_2_	Adiponitrile	4.0
2	110.08439	110.08440	*0.00*	C_6_H_10_N_2_	2-Isopropylimidazole	3.0
3	168.12352	168.12626	−*2.74*	C_9_H_16_N_2_O	3-(1-Ethyl-1-methylpropyl)-5-isoxazolamine	3.0
4	169.12916	169.12962	−*0.45*	C_8_^13^CH_16_N_2_O	Unknown	3.0
5	195.12322	195.12459	−*1.36*	C_9_H_14_N_4_O	Cinachyrazole C	4.5
6	196.12730	196.12794	−*0.64*	C_8_^13^CH_15_N_4_O	Unknown	4.5
7	212.14524	212.14392	*1.31*	C_15_H_18_N	Cadalene	7.5
8	213.15411	213.15175	*2.36*	C_15_H_19_N	8-Hexylisoquinoline	7.0
9	232.19781	232.19395	*3.86*	C_15_H_24_N_2_	1,5-Dicyclohexylimidazole	5.0
10	233.20650	233.20177	*4.73*	C_15_H_25_N_2_	Unknown	4.5
11	234.21224	234.20960	*2.64*	C_15_H_26_N_2_	Sparteine	4.0
12	235.21931	235.21742	*1.89*	C_15_H_27_N_2_	4-Methyl-2-undecylimidazole	3.5
13	236.22711	236.22525	*1.87*	C_15_H_28_N_2_	2-Dodecyl-1H-imidazole	3.0
14	237.22944	237.22860	*0.83*	C_14_^13^CH_28_N_2_	Unknown	3.0
15	239.15370	239.15482	−*1.12*	C_16_H_19_N_2_	4-(4-Dimethylaminostyryl)-1-methylpyridinium	8.5
16	240.15655	240.15818	−*1.63*	C_15_^13^CH_19_N_2_	Unknown	8.5
17	247.18814	247.18959	−*1.45*	C_11_H_25_N_3_O_3_	2-Amino-*N*,*N*-diethylpropanamide; methyl (2S)-2-aminopropanoate	1.0
18	248.19482	248.19295	*1.87*	C_10_^13^CH_25_N_3_O_3_	Unknown	1.0
19	249.20279	249.20524	−*2.45*	C_11_H_27_N_3_O_3_	*N*'-[2-(4,4,4-Trimethoxybutylamino)ethyl]ethane-1,2-diamine	0.0
20	250.20532	250.20860	−*3.27*	C_10_^13^CH_27_N_3_O_3_	Unknown	0.0
21	256.17922	256.18137	−*2.15*	C_16_H_22_N_3_	2-(3-Propylbenzimidazol-3-ium-1-yl)hexanenitrile	7.5
22	257.18411	257.18473	−*0.62*	C_15_^13^CH_22_N_3_	Unknown	7.5

**Table 3:** Constituents identified in *R. raetam* plant stem extract by GC-MS.

S. no.	Proposed compound	Formula	M.W.	Rt (min.)	Area (%)	Composition (%)
1	2,4-di-tert-butylphenol	C_14_H_22_O	206	19.68	0.820	14.584
2	Sparteine	C_15_H_26_N_2_	234	25.93	3.232	7.84
3	Benzenepropanoic acid, 3,5-bis(1,1-dimethylethyl)-4-hydroxy-, methyl ester	C_18_H_28_O_3_	292	28.70	13.460	4.47
4	Phthalic acid, butyl nonyl ester	C_21_H_32_O_4_	348	29.35	4.280	6.89
5	1-Octadecanol	C_18_H_38_O	270	31.62	2.340	4.13
6	5,6-Dehydrolupanine	C_15_H_22_N_2_O	246	34.05	2.321	8.74
7	Diisooctyl phthalate	C_24_H_38_O_4_	390	39.28	36.420	2.13
8	1,3-Benzenedicarboxylic acid, bis(2-ethylhexyl)ester	C_24_H_38_O_4_	390	42.38	4.262	10.54
9	Squalene	C_30_H_50_	410	43.16	28.210	8.91
10	Argentamin	C_15_H_20_N_2_O_2_	260	43.92	1.252	5.20
11	Hentriacontane	C_31_H_64_	436	47.08	0.962	24.71
12	Methyl 8,14-epoxy-15-hydroxy-16-nor-pimarate	C_20_H_32_O_4_	336	49.59	1.587	1.56

**Table 4:** Elements and heavy metals concentration *R. raetam* stem (mgKg^−1^).

Element	Amount (mg Kg^−1^)	Element	Amount (mg Kg^−1^)	Element	Amount (mg Kg^−1^)
Li	1.213 ± 1.6	Mn	108.878 ± 2.7	Ag	0.307 ± 2.4
Na	531.674 ± 2.4	Fe	1054.487 ± 1.0	Cd	0.124 ± 3.0
B	157.829 ± 2.4	Co	0.813 ± 1.1	Sn	1.005 ± 1.8
Mg	7387.130 ± 1.0	Ni	4.508 ± 1.1	Sb	0.1083.1 ± 1.4
Al	409.554 ± 1.1	Cu	6.648 ± 0.7	Ba	15.622 ± 1.0
Si	1122.7 ± 3.0	Zn	17.495 ± 2.1	La	0.456 ± 2.5
P	701.293 ± 2.1	As	1.400 ± 2.4	Ce	0.851 ± 0.5
K	15267.514 ± 0.9	Br	35.389 ± 0.9	W	0.550 ± 0.7
Ca	71951 ± 3.1	Sr	361.079 ± 2.8	Hg	0.299 ± 1.0
Ti	14.276 ± 2.4	Mo	1.049 ± 0.7	Pb	0.960 ± 0.6
Cr	50.962 ± 1.9	Pd	0.279 ± 1.6	Bi	0.406 ± 2.2

**Table 5:** Antimicrobial activities of *R. raetam* plant extract measured by agar well diffusion and MIC methods.

Test microorganisms	Plant extract (mm)	Control (mm)	Plant extract MIC (mm)	Control MIC (mm)
*Salmonella typhi*	3.5	1.3	1.6	6.0
*Escherichia coli*	3.8	2.8	9.3	10.0
*Pseudomonas aeruginosa*	6.2	1.7	2.0	6.5
*Klebsiella pneumoniae*	4.2	2.6	9.2	10.3
*Staphylococcus aureus*	5.8	3.7	5.0	10.5

## Data Availability

All the data included are present within the text.

## References

[1] Nam S., Jang H. W., Shibamoto T. (2012). Antioxidant activities of extracts from teas prepared from medicinal plants, morus alba L., camellia sinensis L., and cudrania tricuspidata, and their volatile components. *Journal of Agricultural and Food Chemistry*.

[2] Kim H. W., Choi S. Y., Jang H. S., Ryu B., Sung S. H., Yang H. (2019). Exploring novel secondary metabolites from natural products using pre-processed mass spectral data. *Scientific Reports*.

[3] Shakya A. K. (2016). Medicinal plants: future source of new drugs. *International Journal of Herbal Medicine*.

[4] Zoubir B., Meriem K. H. (2012). Evaluation of genetic diversity in three species of Retama genus: R. monosperma (L) Boiss, R. raetam (Forssk) Webb and R. sphaerocarpa (L) Boiss.(Fabaceae) based on SDS-PAGE. *Current Research Journal of Biological Sciences*.

[5] Greuter W., Burdet H. M., Long G. (1989). *A Critical Inventory of Vascular Plants of the Circum-Mediterranean Countries*.

[6] Rebbas K., Bounar R., Gharzouli R., Ramdani M., Djellouli Y., Alatou D. (2012). Plantes d’intérêt médicinale et écologique dans la région d’Ouanougha (M’sila, Algérie). *Phytothérapie*.

[7] Nelly A., Annick D. D., Frederic D. (2008). Plants used as remedies antirheumatic and antineuralgic in the traditional medicine of Lebanon. *Journal of Ethnopharmacology*.

[8] Tahraoui A., El-Hilaly J., Israili Z. H., Lyoussi B. (2007). Ethnopharmacological survey of plants used in the traditional treatment of hypertension and diabetes in south-eastern Morocco (Errachidia province). *Journal of Ethnopharmacology*.

[9] Hayet E., Maha M., Samia A. (2008). Antimicrobial, antioxidant, and antiviral activities of Retama raetam (Forssk.) Webb flowers growing in Tunisia. *World Journal of Microbiology and Biotechnology*.

[10] Edziri H., Mastouri M., Mahjoub M. A., Mighri Z., Mahjoub A., Verschaeve L. (2012). Antibacterial, antifungal and cytotoxic activities of two flavonoids from Retama raetam flowers. *Molecules*.

[11] El-Toumy S., Farrag A., Ellithey M., Korien K. (2009). Effect of plant derived-phenolic extracts on antioxidant enzyme activity and mucosal damage caused by indomethacin in rats. *Planta Medica*.

[12] Bremner P., Rivera D., Calzado M. A. (2009). Assessing medicinal plants from South-Eastern Spain for potential anti-inflammatory effects targeting nuclear factor-Kappa B and other pro-inflammatory mediators. *Journal of Ethnopharmacology*.

[13] González-Mauraza H., Martín-Cordero C., Alarcón-de-la-Lastra C., Rosillo M. A., León-González A. J., Sánchez-Hidalgo M. (2014). Anti-inflammatory effects of Retama monosperma in acute ulcerative colitis in rats. *Journal of Physiology and Biochemistry*.

[14] Algandaby M. M., Alghamdi H. A., Ashour O. M. (2010). Mechanisms of the antihyperglycemic activity of Retama raetam in streptozotocin-induced diabetic rats. *Food and Chemical Toxicology*.

[15] Eddouks M., Maghrani M., Louedec L., Haloui M., Michel J. B. (2008). Antihypertensive activity of the aqueous extract of retama raetam forssk. Leaves in spontaneously hypertensive rats. *Journal Of Herbal Pharmacotherapy*.

[16] Maghrani M., Zeggwagh N.-A., Haloui M., Eddouks M. (2005). Acute diuretic effect of aqueous extract of *Retama raetam* in normal rats. *Journal of Ethnopharmacology*.

[17] López-Lázaro M., Martín-Cordero C., Ayuso M. (1999). Flavonoids ofRetama sphaerocarpa. *Planta Medica*.

[18] Kassem M. E., Mosharrafa S. A., Saleh N. A., Wahab S. A. (2006). Flavonoids of *retama raetam*, and in vitro antitumor screening of two isoflavones, *Egypt*. *Journal of Pharmaceutical Sciences*.

[19] El-Shazly A., Ateya A.-M., Witte L., Wink M. (1996). Quinolizidine alkaloid profiles of *Retama raetam, R. sphaerocarpa and R. monosperma*. *Zeitschrift für Naturforschung C*.

[20] Sandberg F. (1958). The alkaloids of *Retama raetam* Webb & berth. *Pharmaceutisch Weekblad*.

[21] González-Mauraza N. H., León-González A. J., Espartero J. L., Gallego-Fernández J. B., Sánchez-Hidalgo M., Martin-Cordero C. (2016). Isolation and quantification of pinitol, a bioactive cyclitol, in retama spp. *Natural Product Communications*.

[22] El Bahri L., Djegham M., Bellil H. (1999). *Retama raetam* W: a poisonous plant of North Africa. *Veterinary and Human Toxicology*.

[23] Mittler R., Merquiol E., Hallak-Herr E., Rachmilevitch S., Kaplan A., Cohen M. (2001). Living under a ’dormant’ canopy: a molecular acclimation mechanism of the desert plant Retama raetam. *The Plant Journal*.

[24] Archer S., Pyke D. A. (1991). Plant-animal interactions affecting plant establishment and persistence on revegetated rangeland. *Journal of Range Management*.

[25] Izhaki I., Ne’eman G. (1997). Hares (Lepusspp.) as seed dispersers ofRetama raetam(Fabaceae) in a sandy landscape. *Journal of Arid Environments*.

[26] Ahamad S. R., Al-Ghadeer A. R., Ali R., Qamar W., Aljarboa S. (2017). Analysis of inorganic and organic constituents of myrrh resin by GC-MS and ICP-MS: an emphasis on medicinal assets. *Saudi Pharmaceutical Journal*.

[27] Frankowski M. (2016). Simultaneous determination of inorganic and organic ions in plant parts of Betula pendula from two different types of ecosystems (Wielkopolski National Park and Chemical Plant in Luboń, Poland). *Environmental Science and Pollution Research*.

[28] Topi D. (2020). Volatile and chemical compositions of freshly squeezed sweet lime (Citrus limetta) juices. *Journal of Raw Materials to Processed Foods*.

[29] Hussain T., Roohi A., Munir S. (2013). Biochemical characterization and identification of bacterial strains isolated from drinking water sources of Kohat, Pakistan. *African Journal of Microbiology Research*.

[30] Biemer J. J. (1973). Antimicrobial susceptibility testing by the Kirby-Bauer disc diffusion method, *Annals Clin*. *Clinical Laboratory Science*.

[31] Trinh P.-C., Thao L.-T.-T., Ha H.-T.-V., Nguyen T. (2020). DPPH-scavenging and antimicrobial activities of Asteraceae medicinal plants on uropathogenic bacteria. *Evidence-Based Complementary and Alternative Medicine*.

[32] Charles P., Kali A., Srirangaraj S. (2016). Comparison of carbapenem breakpoints in clinical laboratory standard Institute and European committee on antimicrobial susceptibility testing guidelines on antibiotic susceptibility test reporting of acinetobacter baumannii. *Indian Journal of Pathology & Microbiology*.

[33] Brand-Williams W., Cuvelier M. E., Berset C. (1995). Use of a free radical method to evaluate antioxidant activity. *LWT - Food Science and Technology*.

[34] Senges J., Ehe L. (1973). Antiarrhythmic action of sparteine on direct and indirect models of cardiac fibrillation. *Naunyn-Schmiedeberg’s Archives of Pharmacology*.

[35] Gremigni P., Hamblin J., Harris D. Genotype× environment interactions and lupin alkaloids. InLupin, an ancient crop for the new millennium.

[36] Paolisso G., Nenquin M., Schmeer W., Mathot F., Meissner H. P., Henquin J. C. (1985). Sparteine increases insulin release by decreasing the K+ permeability of the B-cell membrane. *Biochemical Pharmacology*.

[37] Sgambato S., Paolisso G., Passariello N., Varricchio M., D’Onofrio F. (1987). Effect of sparteine sulphate upon basal and nutrient-induced insulin and glucagon secretion in normal man. *European Journal of Clinical Pharmacology*.

[38] Schmeller T., Wink M. (1998). Utilization of alkaloids in modern medicine.

[39] De la Vega R., Gutierrez M. P., Sanz C. (1996). Bactericide-like effect of Lupinus alkaloids. *Industrial Crops and Products*.

[40] Kim S.-J., Chung W.-S., Kim S.-S., Ko S.-G., Um J.-Y. (2011). Antiinflammatory effect of oldenlandia diffusa and its constituent, hentriacontane, through suppression of caspase-1 activation in mouse peritoneal macrophages. *Phytotherapy Research*.

[41] Khajuria V., Gupta S., Sharma N. (2017). Anti-inflammatory potential of hentriacontane in LPS stimulated RAW 264.7 cells and mice model. *Biomedicine & Pharmacotherapy*.

[42] Nur-e-Alam M., Yousaf M., Parveen I. (2019). New flavonoids from the Saudi Arabian plant Retama raetam which stimulates secretion of insulin and inhibits *α*-glucosidase. *Organic & Biomolecular Chemistry*.

[43] Edziri H., Mastouri M., Chéraif I., Aouni M. (2010). Chemical composition and antibacterial, antifungal and antioxidant activities of the flower oil ofRetama raetam(Forssk.) Webb from Tunisia. *Natural Product Research*.

[44] Colotti G., Ilari A., Boffi A., Morea V. (2013). Metals and metal derivatives in medicine. *Mini-Reviews in Medicinal Chemistry*.

[45] Bauer D. C. (2013). Calcium supplements and fracture prevention. *New England Journal of Medicine*.

[46] Awen B. Z. S., Unnithan C. R., Ravi S. (2011). Essential oils ofRetama raetamfrom Libya: chemical composition and antimicrobial activity. *Natural Product Research*.

